# Natural rubber as a renewable carbon source for mesoporous carbon/silica nanocomposites

**DOI:** 10.1038/s41598-020-69963-3

**Published:** 2020-07-31

**Authors:** Satit Yousatit, Hannarong Pitayachinchot, Apinya Wijitrat, Supphathee Chaowamalee, Sakdinun Nuntang, Siriwat Soontaranon, Supagorn Rugmai, Toshiyuki Yokoi, Chawalit Ngamcharussrivichai

**Affiliations:** 1 Department of Chemical Technology, Faculty of Science, Chulalongkorn University, Pathumwan, Bangkok, 10330 Thailand; 2 Center of Excellence in Catalysis for Bioenergy and Renewable Chemicals (CBRC), Faculty of Science, Chulalongkorn University, Pathumwan, Bangkok, 10330 Thailand; 3 Center of Excellence on Petrochemical and Materials Technology (PETROMAT), Chulalongkorn University, Pathumwan, Bangkok, 10330 Thailand; 4 Industrial Chemistry and Textile Technology Programme, Faculty of Science, Maejo University, Chiang Mai, 50290 Thailand; 5 Synchrotron Light Research Institute (SLRI), Nakhon Ratchasima, 30000 Thailand; 6 Nanospace Catalysis Unit, Innovative Research Institute, Tokyo Institute of Technology, 4259 Nagatsuta, Midori-ku, Yokohama, 226-8503 Japan

**Keywords:** Chemistry, Engineering, Materials science

## Abstract

This study is the first report on the preparation of mesoporous carbon/silica (MCS) nanocomposites with tunable mesoporosity and hydrophobicity using natural rubber (NR) as a renewable and cheap carbon source. A series of mesoporous nanocomposites based on NR and hexagonal mesoporous silica (HMS) were prepared via an in situ sol–gel process and used as precursors; then, they were converted into MCS materials by controlled carbonization. The NR/HMS precursors exhibited a high dispersion of rubber phase incorporated into the mesostructured silica framework as confirmed by small-angle X-ray scattering and high-resolution transmission electron microscopy. An increase in the carbonization temperature up to 700 °C resulted in MCS nanocomposites with a well-ordered mesostructure and uniform framework-confined wormhole-like channels. The NR/HMS nanocomposites possessed high specific surface area (500–675 m^2^ g^−1^) and large pore volume (1.14–1.44 cm^3^ g^−1^). The carbon content of MCS (3.0–16.1 wt%) was increased with an increase in the H_2_SO_4_ concentration. Raman spectroscopy and X-ray photoelectron spectroscopy revealed the high dispersion of graphene oxide-like carbonaceous moieties in MCS materials; the type and amount of oxygen-containing groups in obtained MCS materials were determined by H_2_SO_4_ concentration. The enhanced hydrophobicity of MCS nanocomposites was related to the carbon content and the depletion of surface silanol groups, as confirmed by the water sorption measurement. The study on the controlled release of diclofenac in simulated gastrointestinal environment suggests a potential application of MCS materials as drug carriers.

## Introduction

Mesoporous carbon/silica nanocomposites (MCS) have received considerable attention in catalysis^1^, adsorption^2^, energy storage^3,4^, and drug delivery^5^ owing to the combined advantages of inorganic silica and organic carbon in their mesostructure. Silica framework provides high mesoporosity, specific surface area, and thermal/mechanical stability. Due to the high density of silanol groups, the silica surface can be simply modified by either direct co-condensation or postsynthesis grafting to acquire various chemically active functionalities to serve a wide range of applications^6,7^. However, amorphous carbon is characterized by its tunable physicochemical properties by controlling the ratio of sp^2^/sp^3^ bonds and quantity of heteroatoms (i.e., oxygen)^8,9^. The oxygen-containing functional groups on the carbon surface provide acidic (i.e., carboxyl, lactone, and phenol) and basic (i.e., pyrone, chromene, ether, and carbonyl) properties^10^. An increase in the amount of sp^2^-hybridized carbon transforms amorphous carbon into graphite-like carbon with enhanced textural properties and chemical reactivity^11,12^. Typically, the preparation of MCS materials consists of two steps. The first step is to introduce an organic substance (e.g., glucose, furfuryl alcohol, phenol, and formaldehyde) as a carbon source into mesostructured silica. Then, the organic substance is converted to carbon by carbonization or hydrothermal treatment^1–4,13–15^.

Mesoporous nanocomposites comprised of natural rubber (NR), as a hydrophobicity improver, dispersed in the wormhole-like mesostructure of hexagonal mesoporous silica (HMS) have been previously synthesized by an in situ sol–gel process using dodecylamine (DDA) as organic template^16–18^. The NR/HMS materials exhibited good structural and textural properties (high specific surface area and large pore volume) owing to the well-dispersed rubber phase in mesostructured silica^16^. Recently, it has been reported that the mesoporosity and hydrophobicity of NR/HMS materials can be systematically controlled by altering the hydrocarbon chain length of amine templates^18^. The surface silanol groups remaining on the silica phase allowed nanocomposites to be modified with different types of chemically active functional groups to prepare hydrophobic mesoporous materials for adsorption^19^ and catalysis^20,21^. However, the major disadvantages of rubber component in mesostructured NR/HMS are poor thermal stability and poor resistance to organic solvents. This reason has motivated us to improve the physicochemical properties of NR/HMS nanocomposites by converting rubber phase into carbonaceous moieties that are highly dispersed in mesostructured silica. The resulting materials are a new class of MCS nanocomposites.

Diclofenac is a nonsteroidal anti-inflammatory drug (NSAID) of a phenylacetic acid derivative with analgesic and antipyretic properties. It has been extensively used to relieve menstrual pain, migraines, and arthritis pain^22^. However, the biological half-life of diclofenac was very short (only 1–2 h), which led to the propensity of systemic accumulation. The long-term excess of diclofenac causes serious side effects such as gastrointestinal and renal dysfunction^23^. Organic/silica composites are attractive carriers for the sustained release of diclofenac in simulated gastrointestinal environment owing to their high specific surface area and porosity, tunable surface properties, and good biocompatibility^24–27^. This drug delivery system maintained adequate concentrations of diclofenac for a specific period of time^28^, which reduced side effects in the stomach and increase the effective biological half-life^29^.

In this study, we report a new use of NR, a renewable and inexpensive polymer, contained in the NR/HMS nanocomposites as a carbon precursor for the preparation of MCS materials by controlled carbonization. The effects of carbonization temperature, concentration of sulfuric acid (H_2_SO_4_) solution used in the pretreatment step, and initial NR content of the NR/HMS precursor on the physicochemical properties of obtained MCS nanocomposites were investigated. The MCS materials were characterized by a well-ordered mesostructure, high mesoporosity, and enhanced hydrophobicity. To verify their potential application, we conducted a preliminary study of representative MCS material in the controlled release of diclofenac in the simulated gastrointestinal solution. To our knowledge, this work is the first report on the preparation of MCS nanocomposites using NR as a carbon source and their application as drug carrier.

## Results and discussion

### Strategic approach for NR/HMS conversion into MCS nanocomposites

The distribution of organic carbon precursors in mesostructured silica is a crucial factor to prepare MCS materials with good structural and textural properties via the carbonization process. The encapsulation of organic substance in mesoporous silica channels via wet impregnation^1,2^ and chemical functionalization of mesostructured silica surface with organosilanes or organic compounds^4,13^ resulted in the poor distribution of carbon phase in obtained nanocomposites. However, mesostructured organic–inorganic nanocomposites prepared using the evaporation-induced co-assembly method in the presence of phenolic resin-based precursors^15^ provided MCS nanocomposites with high carbon content and well-dispersed carbon moieties^15^ due to the high dispersion of the polymeric carbon precursor in the mesostructured silicate framework.

**Figure 1:**
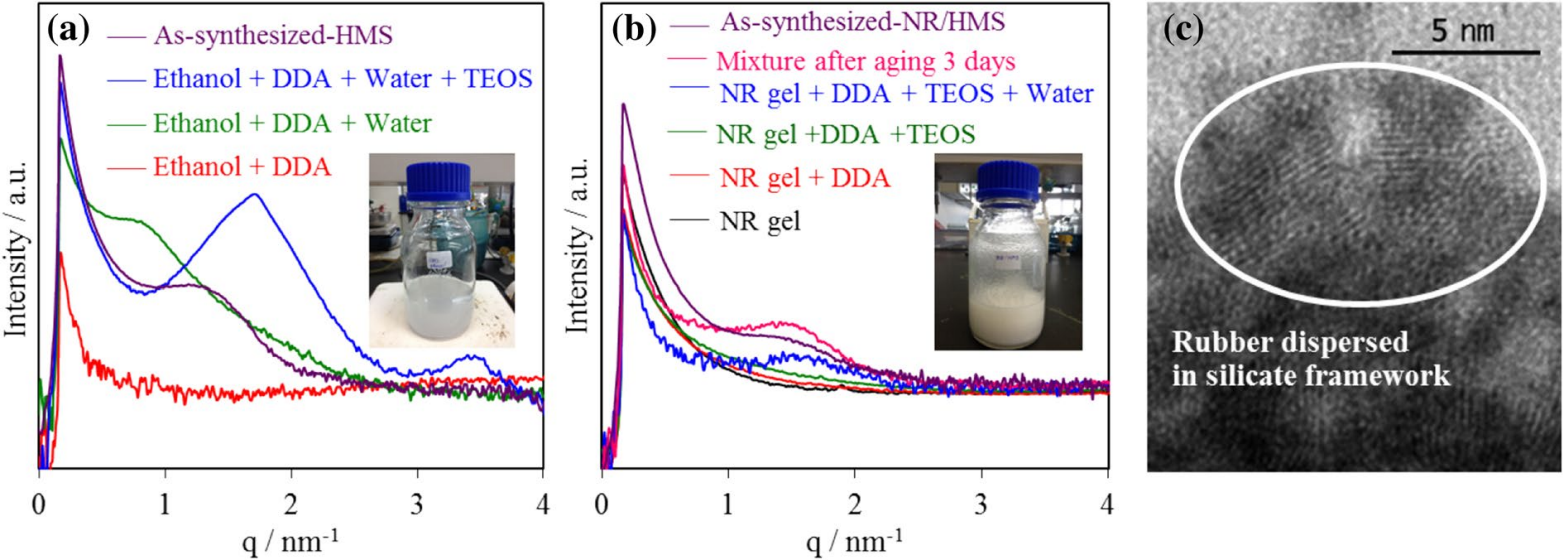
Representative SAXS patterns of the preparation of as-synthesized (**a**) HMS and (**b**) NR/HMS with different compositions, and (**c**) representative HRTEM image (×400,000 magnification) of NR/HMS stained with a 1-wt% OsO_4_ aqueous solution; inset photos in (**a**) and (**b**) are the mother gels of HMS and NR/HMS, respectively, prior to aging.

**Figure 2:**
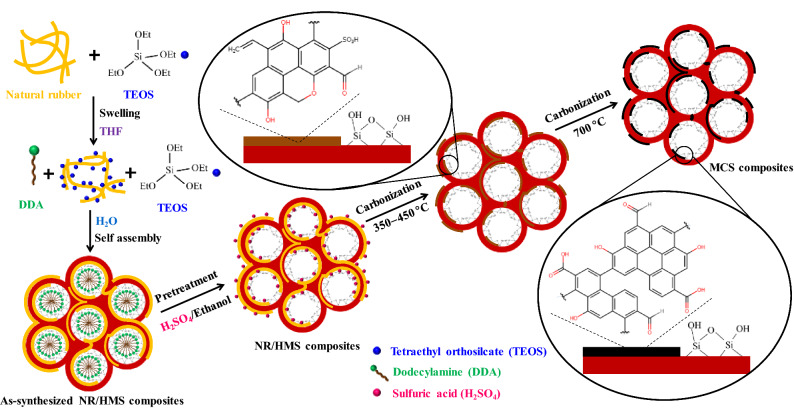
Schematic diagram of the mechanistic model for the formation of NR/HMS nanocomposites and their transformation to MCS materials.

In this study, the incorporation and distribution of rubber phase in the mesostructured silicate framework of NR/HMS were confirmed by small-angle X-ray scattering (SAXS) and high-resolution transmission electron microscopy (HRTEM), respectively. Figure 1 compares the SAXS patterns of organic–inorganic mesophases in the mixtures during the synthesis of HMS and NR/HMS. For NR/HMS (Fig. 1a), the peak at q = 1.46 nm^−1^, which corresponds to the mesophase structure with a hexagonal unit cell of 0.68 nm, was observed when the rubber gel was mixed with tetraethyl orthosilicate (TEOS), dodecylamine (DDA), and water. It was larger than the mesophase formed during the synthesis of pure silica HMS (unit cell = 0.58 nm) with the peak at q = 1.71 nm^−1^ (Fig. 1b). The obtained result indicates that rubber molecules were incorporated into the organic–inorganic mesophase of NR/HMS. Figure 2 indicates the proposed mechanistic model for the formation of NR/HMS nanocomposites synthesized via the in situ sol–gel process. First, TEOS and DDA were homogeneously dissolved in the NR solution using tetrahydrofuran as the synthesis media. Upon the addition of H_2_O, TEOS was partially hydrolyzed to silicate species simultaneously with the rearrangement of DDA molecules into hexagonal rod-like micelles, induced by H-bonding between the amine groups of DDA and hydroxyl groups of silicate species. The ethoxy groups adjacent to rubber molecules remained nonhydrolyzed and acted as linkers between the rubber chains and silicate oligomers, which resulted in mesostructured entrapped NR/silica composite framework. The HRTEM image indicates the high dispersion of rubber lamellae in the NR/HMS nanocomposite obtained by this approach (Fig. 1c).

**Figure 3:**
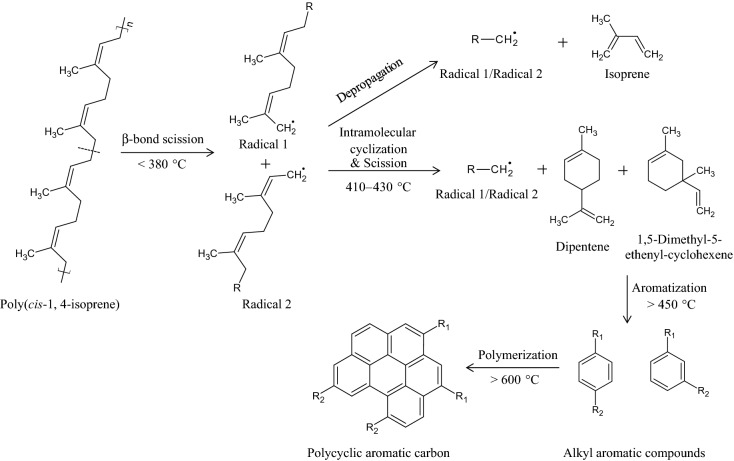
Possible mechanism for the degradation and transformation of poly(*cis*-1,4-isprene) into polycyclic aromatic carbons.

Our strategy for the conversion of the NR/HMS precursor into MCS composites via carbonization is shown in Fig. 2. In the first step, the as-synthesized NR/HMS nanocomposite was pretreated with an H_2_SO_4_/ethanol solution. H_2_SO_4_ adsorbed on the NR/HMS precursor was supposed to work as a catalyst to convert rubber phase into carbonaceous residue during the subsequent carbonization process. The thermal degradation of NR, a polymer of *cis* 1,4-isoprene, is a radical reaction, which is initiated by the random-chain scission of the β bond with respect to the double bonds of the polymeric backbone^30^. As shown in Fig. 3, two different allylic radicals are generated at temperatures below 380 °C, which results in lower molecular weight polyisoprene^31^. At higher temperatures (410–430 °C), both radicals undergo depropagation or unzipping toward isoprene monomers, simultaneously with intramolecular cyclization, followed by scission, to yield dipentene and other cycloalkene derivatives^30,32,33^. A further increase in the temperature to > 450 °C enhances fragmentation and aromatization of formed products, affording isoprene, terpenes, and aromatic compounds such as *p*-cymene^34^. In the presence of acid catalysts, the yield of aromatics was increased^35^, and the crude products from NR degradation could be repolymerized^31^. Therefore, the content and chemical nature of carbon moieties formed in the resulting MCS materials should be determined by carbonization temperature, concentration of H_2_SO_4_ solution used in the pretreatment step, and initial NR content of the NR/HMS precursor.

### Effect of carbonization temperatures on MCS formation

**Figure 4:**
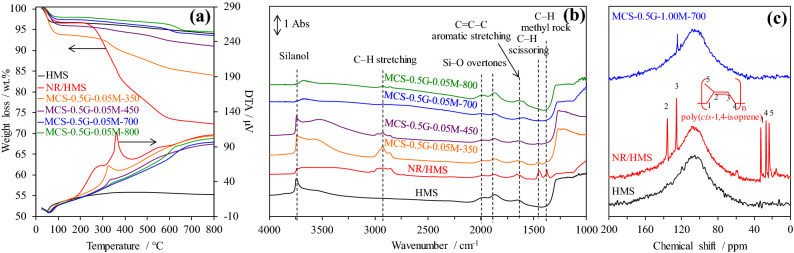
Representative (**a**) weight loss and DTA curves, and (**b**) FT-IR spectra of HMS, NR/HMS, and MCS-0.5G-0.05M-*x* series prepared at different carbonization temperatures; Representative (**c**) solid-state ^13^C NMR spectra of HMS, NR/HMS, and MCS-0.5G-1.00M-700.

The weight loss and differential thermal analysis (DTA) curves of HMS, NR/HMS, and MCS nanocomposites obtained at different carbonization temperatures are compared in Fig. 4a. The NR/HMS showed the major weight loss of ~ 17 wt% at 150–420 °C owing to the decomposition of NR incorporated into the mesostructured silicate framework. The small weight loss (~ 7 wt%) in the range of 420–650 °C was related to the rubber-derived carbon residue^16^ and dehydroxylation of silicate network^36^. Some rubber fractions still remained in the resulting nanocomposites obtained at 350 °C. This decomposition step disappeared at the carbonization temperature of 450 °C. As shown in Table 1, the carbon content of the MCS nanocomposites prepared at 700 °C and 800 °C was insignificantly different (3.4–3.5 wt%) and not affected by increased carbonization temperature. This result was similar to that of Kim et al.^37^, who observed an insignificant change in the carbon content of carbon materials prepared from cellulose and carbonized at 500–800 °C. In general, the majority of weight loss for polymers up to 500 °C was due to the release of volatile matter, after which the structure of carbon residue was rearranged from amorphous carbon toward a more crystalline phase at higher temperatures^38^.

**Table 1:** Physicochemical properties of pure silica HMS, NR/HMS precursor, and MCS nanocomposites prepared under different conditions.

Sample	Carbon content^b^ (wt.%)	*S*_BET_^c^ (m^2^ g^−1^)	*S*_ext_^d^ (m^2^ g^−1^)	*D*_p_^e^ (nm)	*V*_t_^f^ (cm^3^ g^−1^)	*V*_p_^g^ (cm^3^ g^−1^)	*d*_100_^h^ (nm)	*a*_0_^i^ (nm)	*W*_t_^j^ (nm)	*V*_m_^*k*^ (cm^3^ g^−1^)
HMS	n.d.	815	216	2.94	1.80	0.50	4.80	5.55	2.61	82.9
NR/HMS^a^	23.9	589	323	2.35	1.40	0.16	5.08	5.87	3.52	40.1
MCS-0.5G-0.05M-350	9.1	737	399	2.42	1.70	0.23	4.80	5.55	3.13	n.d
MCS-0.5G-0.05M-450	4.2	766	450	2.44	1.73	0.24	4.75	5.49	3.05	n.d
MCS-0.5G-0.05M-700	3.4	675	387	2.40	1.44	0.15	4.91	5.67	3.27	41.6
MCS-0.5G-0.05M-800	3.3	338	184	1.80	0.93	0.07	n.d	n.d	n.d	n.d
MCS-0.5G-0.50M-700	4.3	670	342	2.37	1.42	0.21	4.91	5.67	3.30	35.2
MCS-0.5G-1.00M-700	10.4	664	322	2.37	1.41	0.19	4.97	5.73	3.36	22.0
MCS-0.5G-1.50M-700	13.1	524	312	2.24	1.18	0.11	5.02	5.80	3.56	34.4
MCS-0.5G-2.00M-700	16.1	500	316	2.12	1.14	0.10	5.08	5.87	3.75	45.2
MCS-1.0G-1.00M-700	10.2	631	311	2.24	1.35	0.17	4.97	5.73	3.49	23.5
MCS-1.5G-1.00M-700	10.8	602	287	2.22	1.30	0.17	4.97	5.73	3.41	22.0

*n.d.* not determined.

^a^Extracted in a 0.05 M H_2_SO_4_/ethanol solution.

^b^Determined by TGA.

^c^BET surface area.

^d^External surface area determined from *t*-plot curves.

^e^Pore diameter calculated using the BJH method.

^f^Total pore volume.

^g^Mesopore volume.

^h^Interplanar spacing of the (100) plane (*d*_100_) obtained from XRD analysis.

^i^The repeat distance (*a*_0_) between pore centers of the hexagonal structure was calculated from *a*_0_ = 2*d*_100_/3^½^

^j^The framework wall thickness was determined by subtracting the BJH mesopore size from the repeat distance between pore centers.

^k^Determined by water adsorption–desorption.

The conversion of the NR phase into carbon residue during the carbonization process was also confirmed by FT-IR (Fig. 4b) and solid-state ^13^C NMR (Fig. 4c). The presence of rubber in the NR/HMS precursor was confirmed by FT-IR bands related to C–H stretching (2,800–3,000 cm^−1^) and C–H deformation vibrations (1,370 cm^−1^ and 1,430 cm^−1^)^16^, the ^13^C-NMR spectrum showed chemical shifts at 23, 27, 33, 127, and 136 ppm, which were attributed to –CH_3_, –CH_2_–, –CH_2_–, =CH–, and > C=, respectively^39–41^, of poly(*cis*-1,4-isoprene) in the NR structure. Compared to pure silica HMS, the presence of rubber in NR/HMS decreased the free silanol band (3,750 cm^−1^) because some remnant ethoxy groups remained in the silicate framework^16,17^. The carbonization at 350–450 °C restored silanol groups, while the characteristic bands of NR gradually decreased with an increase in temperature. Above 700 °C, the band at 1,580–1615 cm^−1^, which corresponded to C=C–C stretching^42^, revealed the presence of aromatic carbon residue in MCS nanocomposites. This observation agreed with a new ^13^C-NMR signal at 128 ppm, which was attributed to aromatic carbon species^7^, observed for MCS-0.5G-1.00M-700. These results indicate the decomposition and transformation of rubber to aromatic carbon during carbonization at 700 °C. Of note, the temperature, at which aromatic carbon species formed in this study, was lower than that used for the preparation of sucrose-derived carbon materials via carbonization at 800 °C^43^.

### Structural and textural properties of MCS materials

**Figure 5:**
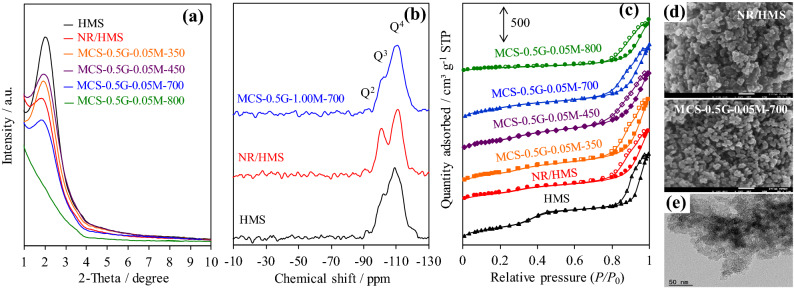
Representative (**a**) XRD patterns of HMS, NR/HMS, MCS nanocomposites with different carbonization temperature; solid-state ^29^Si NMR spectra of (**b**) HMS, NR/HMS, and MCS-0.5G-1.00M-700; N_2_ adsorption–desorption isotherms of (**c**) HMS, NR/HMS, MCS nanocomposites with different carbonization temperature; (**d**) FE-SEM images of NR/HMS and MCS-0.5G-0.05M-700 at ×150,000 magnification. (**e**) TEM images of MCS-0.5G-0.05M-700 at ×100,000 magnification.

The small-angle X-ray diffraction (XRD) patterns of pure silica HMS and representative nanocomposites exhibited an intense reflection at 2*θ* in the range of 1.5°–3.0° (Fig. 5a), which corresponded to the (100) plane of hexagonal mesostructure with a wormhole-like silicate framework^16^. Basically, silica-based materials consisted of Si(OSi)_2_(OH)_2_, Si(OSi)_3_(OH), and Si(OSi)_4_ species, which corresponded to Q^2^ (− 92 ppm), Q^3^ (− 101 ppm), and Q^4^ (− 110 ppm) resonances, respectively, as revealed in the ^29^Si magic angle spinning (MAS) NMR spectra (Fig. 5b). The ratio of Q^2^:Q^3^:Q^4^ species of HMS, NR/HMS, and MCS were 1:7.5:33, 1:11:21, and 1:6.4:36, respectively. The presence of rubber incorporated into the HMS structure enhanced the wall thickness (*W*_t_) of NR/HMS (Table 1) but hampered the hexagonal ordering of mesoporous structure^16–18^. Moreover, the higher content of isolated silanol sites (Q^3^) in NR/HMS than in HMS indicated that rubber molecules present in the synthesis mixture lowered the degree of silicate condensation^17^.

An increase in the carbonization temperature up to 450 °C increased the (001) reflection and led to reduced *W*_t_ and contraction of hexagonal unit cell (*a*_0_) of MCS nanocomposites. The comparison of intensities of Q^*n*^ groups in composites revealed that the obtained MCS had lower Q^2^ and Q^3^ but higher four siloxane-bonded site (Q^4^) than the NR/HMS precursor. The overall result indicated that the carbonization process enhanced the condensation of incompletely hydrolyzed silicate species to form siloxane bonds^44^. Although MCS-0.5G-0.05M-700 exhibited the loss of mesostructure ordering owing to the dehydroxylation of silicate networks, which was enhanced at > 500 °C^45,46^, its *a*_0_ and *W*_t_ values increased compared to those of HMS calcined at 700 °C (Table 1). This result suggests that the formed carbon layer acted as a rigid support in the wall and probably limited structural shrinkage. This result was similar to that reported by Liu et al.^15^, who observed an interpenetrating network of carbon–silica nanocomposites prepared from a soluble resol polymer as a carbon source via evaporation-induced triconstituent co-assembly. The carbon layer acted as a rigid support and decreased framework shrinkage. Unfortunately, the mesostructured framework of MCS-0.5G-0.05M-800 collapsed due to severe dehydroxylation.

The N_2_ adsorption–desorption isotherms revealed that both pure silica HMS and nanocomposites exhibited type IV sorption isotherms (Fig. 5c), according to the IUPAC classification, which is characteristic of framework-confined mesoporous materials. NR/HMS exhibited lower textural properties than HMS (Table 1), which suggested that part of rubber molecules might cover the pore mouths or occupy the porous channels of mesostructured silica. This conclusion was supported by the FE-SEM analysis (Fig. 5d), which revealed the presence of particle agglomerates (102–167 nm). Compared to the NR/HMS precursor, the MCS nanocomposites obtained at 350 °C and 450 °C showed enhanced textural properties. Carbonization decomposed rubber covering the precursor surface and enhanced the condensation of silicate framework. The resulting MCS materials possessed reduced particle size and lower aggregation such as MCS-0.5G-0.05M-700 with an average size of 20–32 nm (Fig. 5d). In addition, the representative Transmission electron microscopy (TEM) image of this MCS sample (Fig. 5e) showed conventional uniform wormhole-like mesopores, as observed for HMS materials^16–18^.

### Chemical structure of carbon species in MCS materials

**Figure 6:**
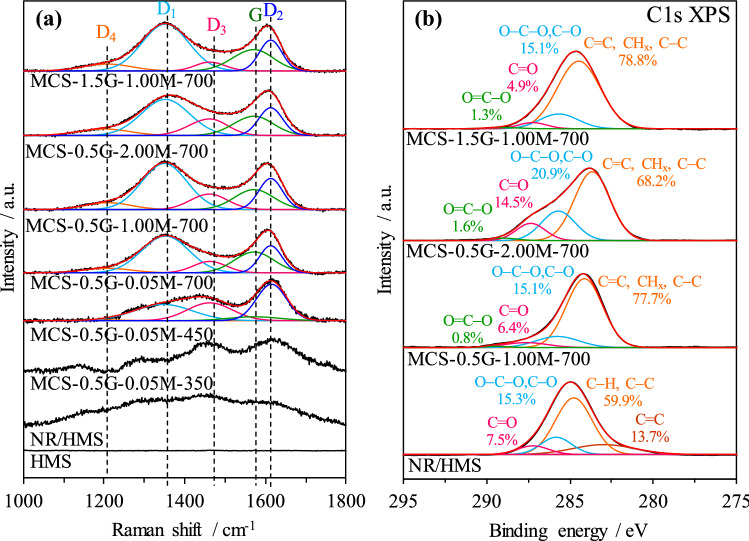
Representative (**a**) Raman spectra of HMS, NR/HMS, MCS-0.5G-0.05M-350, MCS-0.5G-0.05M-450, MCS-0.5G-0.05M-700, MCS-0.5G-1.00M-700, MCS-0.5G-2.00M-700, and MCS-1.5G-1.00M-700. (**b**) C1s XPS spectra of NR/HMS, MCS-0.5G-1.00M-700, MCS-0.5G-2.00M-700, and MCS-1.5G-1.00M-700.

Raman spectroscopy and X-ray photoelectron spectroscopy (XPS) were applied to acquire information about the evolution of the chemical nature of carbon species on MCS nanocomposites. As shown in Fig. 6a, the NR/HMS precursor exhibited a broad band in the range of 1,090–1,780 cm^−1^, which was attributed to the polymeric backbone of rubber incorporated into the HMS structure^47^. It corresponded to the C1s XPS signals (Fig. 6b) of NR/HMS, which represented the C=C bond (282.9 eV) and C–C/C–H bonds (284.8 eV)^17^. The C–O/C–O–C bonds (285.8 eV) and C=O bond (287.2 eV) were the surface functional groups formed by the oxidative cleavage of NR chains during storage^48^. Residual rubber moieties still existed on MCS-0.5G-0.05M-350. A weak band at approximately 1,150 cm^−1^ was attributed to the O=S=O symmetric stretching vibration of the sulfonic acid group grafted on the carbon surface^49,50^. An increase in temperature matured the carbon residue structure, as deduced from increased band intensity.

The resulting MCS nanocomposites exhibited two well-developed bands at approximately 1,370 cm^−1^ (D band) and 1,590 (G band) cm^−1^, which revealed the disorder or defects in the organization of carbon atoms and the sp^2^ in-plane vibration of graphitic carbon atoms, respectively^51^. Moreover, the O=S=O band disappeared at the carbonization temperature of 700 °C, which supported the rearrangement of amorphous carbon residue to graphitic carbon structure^6^. In the high Raman shift region (Fig. S1 in SI), two bands, at 2,650 cm^−1^ (D′_1_ band) and 2,850 cm^−1^ (D′_3_ band), were observed and attributed to the overtone of D or D_1_ and the combination of D and G, respectively^52^. These bands were related to the second order scattering of imperfect graphite and disordered carbons such as graphene oxide-like carbon species^53^. The result agreed with the C1s XPS spectrum of representative MCS materials, which showed carbon groups (C=C, CH_x_, and C–C) at 283.6 eV, hydroxyl groups or ether linkages (C–O, C–O–C) at 285.3 eV, carbonyl groups (C=O) at 287.2 eV, and carboxyl or ester groups (COO) at 289.0 eV. In addition, the O1s XPS spectrum (Fig. S2 in SI) confirmed the presence of carboxyl or ester groups at 530.3 eV, and hydroxyl or ether groups at 531.7 eV^54^. These results suggest that the resulting MCS nanocomposites exhibited the high dispersion of graphene oxide-like carbonaceous moieties with different types of surface oxygen-containing groups (C–O, C=O, C–O–O, and O–C=O).

### Tuning physicochemical properties of MCS materials

**Figure 7:**
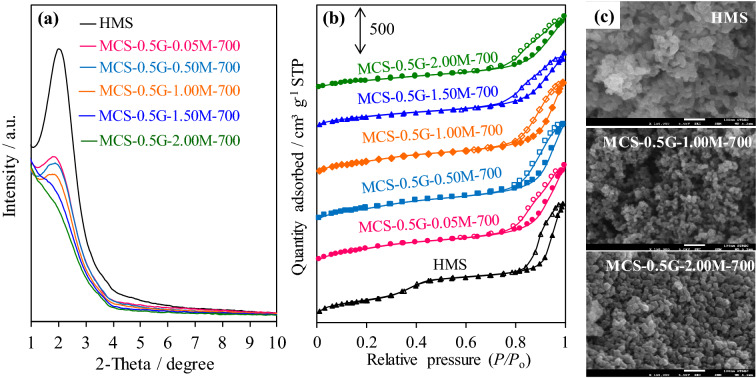
Representative (**a**) XRD patterns and (**b**) N_2_ physisorption isotherms of HMS and MCS nanocomposites prepared using different H_2_SO_4_ concentrations; (**c**) FE-SEM images of HMS, MCS-0.5G-1.00M-700, and MCS-0.5G-2.00M-700 at ×150,000 magnification.

The concentration of H_2_SO_4_ during the pretreatment step and the initial NR content of the NR/HMS precursor were varied to tune the physicochemical properties of resulting MCS nanocomposites. As summarized in Table 1, the carbon residue in the composite structure (3.4–16.1 wt%) was systematically increased with increasing H_2_SO_4_ concentrations from 0.5 to 2.0 M. The XRD patterns of MCS in this series exhibited characteristic peaks at lower 2*θ* positions compared to those of pure silica HMS (Fig. 7a), which corresponded to enhanced *a*_0_ and *W*_t_ values (Table 1), concomitantly with an enlarged particle size (Fig. 7c^),^ owing to an increase in the amount of carbon incorporated into the mesostructured silica framework. However, an increase in the initial NR content of the NR/HMS precursor from 0.5 to 1.5 g did not affect the carbon content and *a*_0_ value of resulting MCS nanocomposites. This result was ascribed to the limited incorporation of NR molecules into the mesostructure of HMS.

The MCS nanocomposites prepared under different conditions exhibited a typical N_2_ physisorption isotherm of mesostructured materials (Fig. 7b). The use of higher H_2_SO_4_ concentration decreased *S*_BET_, *D*_p_, and *V*_t_ (Table 1), which correlated well with the trend in the carbon content of this MCS series because some carbon residue may occupy the mesopores of resulting nanocomposites. By combining this result with XRD analysis confirmed that MCS prepared with a high NR content had a higher carbon phase fraction occluding into the mesopores than MCS prepared from the NR/HMS precursor with high NR dispersion.

Valle-Vigón et al.^6^ have reported that MCS materials prepared using a Pluronic P123 triblock co-polymer template as a carbon source via 800 °C carbonization had the highest carbon content of 13 wt%, *S*_BET_ of 460 m^2^ g^−1^, and *V*_t_ of 0.58 cm^3^ g^−1^. Furthermore, the sucrose-derived MCS^7^, which was prepared by the co-assembly method, containing 16 wt% carbon, exhibited *S*_BET_ and *V*_t_ of 316 m^2^ g^−1^ and 0.82 cm^3^ g^−1^, respectively. This result indicates that MCS nanocomposites prepared from the NR/HMS precursor not only had a high amount of carbon but also superior textural properties compared to previously reported MCS materials with a similar carbon content.

The Raman spectra of MCS materials obtained using different H_2_SO_4_ concentrations and initial NR content of the NR/HMS precursor are compared in Fig. 6a. The D and G bands were deconvoluted into five components, which were assigned to polyenes (D_4_; 1,208 cm^−1^), graphene edges (D_1_; 1,352 cm^−1^), amorphous carbon (D_3_; 1,462 cm^−1^), graphitic carbon (G; 1572 cm^−1^), and graphene sheets (D_2_; 1,610 cm^−1^)^7,52^. The fitting parameters obtained from the Raman analysis are summarized in Table S1 (SI). An increase in the H_2_SO_4_ concentration from 0.05 M to 2.00 M increased the fraction of amorphous carbon (D_3_), whereas the formation of crystalline carbon (G) decreased from 21.9% (MCS-0.5G-0.05M-700) to 18.7% (MCS-0.5G-2.00M-700). The use of high H_2_SO_4_ concentration may result in the high degree of sulfonic acid groups being grafted onto carbon residue^6^, which prevented the rearrangement of amorphous carbon to graphitic carbon. Furthermore, the C1s XPS analysis (Fig. 6b) indicated an increased content of oxygen-containing groups on the carbon surface due to the oxidation of carbonaceous residue by H_2_SO_4_. A similar observation was reported in the sulfonation of biochar using a concentrated H_2_SO_4_ solution, which not only introduced sulfonic acid groups but also generated carboxyl and hydroxyl groups on the resulting acidic carbon^55–57^. The fraction of oxygen-containing groups on MCS nanocomposites prepared from the NR/HMS precursor (21.3–32.0%) was higher than that on MCS materials prepared using furfuryl alcohol as a carbon precursor (12.0%)^58^. An increase in the initial NR content of the NR/HMS precursor from 0.5 to 1.5 g resulted in a greater graphitized carbon phase (G) in resulting MCS nanocomposites (Table S1 in SI).

H_2_SO_4_ enhanced dehydration reactions and the substitution of sulfonic acid groups onto the resulting amorphous carbon at low temperatures^6^. With an increase in the carbonization temperature, the oxygen-containing functional groups decomposed to H_2_O, carbon monoxide, carbon dioxide, and sulfur dioxide^6,59^. Moreover, H_2_SO_4_ was essential in generating carbonaceous residues from NR molecules by promoting the formation of aromatic structures and cross-linking processes^6^. It is worth noting that MCS nanocomposites prepared from the NR/HMS precursor had the highest carbon yield of 67.4 wt%. Nishihara et al.^3^ have observed a 22.9–37.3 wt% carbon yield for MCS materials prepared by coating mesoporous silica SBA-15 with 2,3-dihydroxynaphthalene as a carbon source followed by carbonization at 800 °C. Raman analysis showed that the degree of graphitization of carbon phase [*I*_D1_/(*I*_G_ + *I*_D1_ + *I*_D2_] contained in MCS materials was in the range of 0.53–0.57, which indicated a similarity in their carbon structure. The relatively large fraction of D_1_ (40.75–48.23%) suggested that carbon residue was present as highly dispersed nanosized graphene in mesostructured nanocomposites.

### Hydrophobicity analysis by the H_2_O adsorption measurement

**Figure 8:**
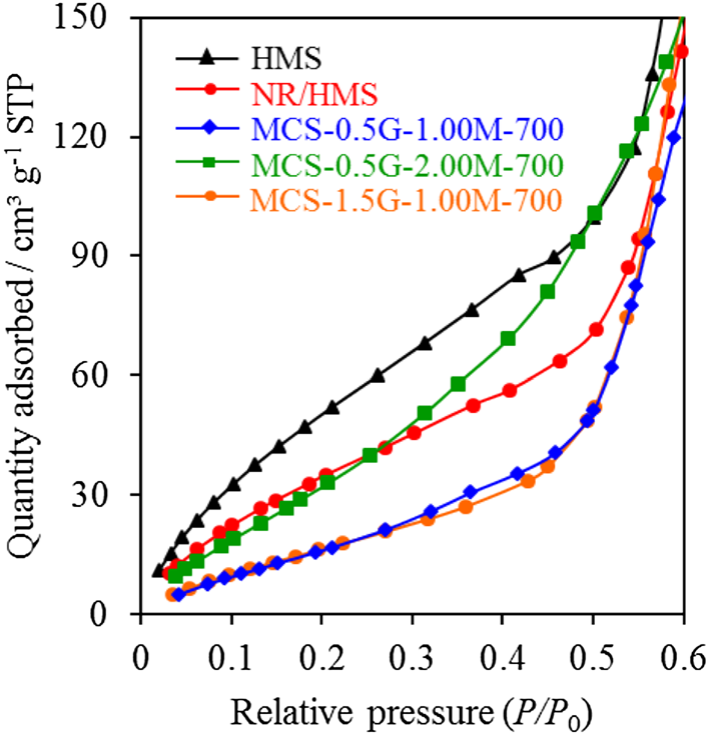
Representative H_2_O adsorption isotherms of (**a**) HMS, (**b**) NR/HMS, (**c**) MCS-0.5G-1.00M-700, (**d**) MCS-0.5G-2.00M-700, and (**e**) MCS-1.5G-1.00M-700 at low relative pressures (*P/P*_*0*_) in the range of 0–0.6

The behavior of H_2_O adsorption at a low relative pressure was used to evaluate the effects of carbonaceous residue on the hydrophobic properties of MCS nanocomposites. Figure 8 showcases the H_2_O adsorption isotherms of samples at *P*/*P*_0_ of 0–0.6. NR/HMS exhibited a lower amount of adsorbed H_2_O than HMS owing to not only the depletion of exposed surface silanol groups, as revealed by the FT-IR analysis, but also the hydrophobic environment created by the rubber phase. Compared with pure silica HMS, MCS materials showed a lower adsorbed volume owing to the depletion of silanol groups via dehydroxylation during the carbonization and hydrophobicity of carbon moieties dispersed in the resulting nanocomposites. Of note, MCS-0.5G-1.0M-700 and MCS-1.5G-1.0M-700 were more hydrophobic than their NR/HMS precursor. An increase in the H_2_SO_4_ concentration to 2.0 M lowered the hydrophobicity of MCS materials because this high acidic condition enhanced the content of oxygen-functional groups on the nanocomposite surface, as evidenced by XPS results. The analysis of adsorption data at a low relative pressure (Table 1) indicated a decreased monolayer adsorption volume (*V*_m_) in the following order: HMS (82.9 cm^3^ g^−1^) > MCS-0.5G-2.00M-700 (45.2 cm^3^ g^−1^) > MCS-0.5G-0.05M-700 (41.6 cm^3^ g^−1^) > NR/HMS (40.1 cm^3^ g^−1^) > MCS-0.5G-1.0M-700 (22.0 cm^3^ g^−1^) ≈ MCS-1.5G-1.0M-700 (22.0 cm^3^ g^−1^).

### Preliminary investigation of HMS and nanocomposites as drug carriers

**Figure 9:**
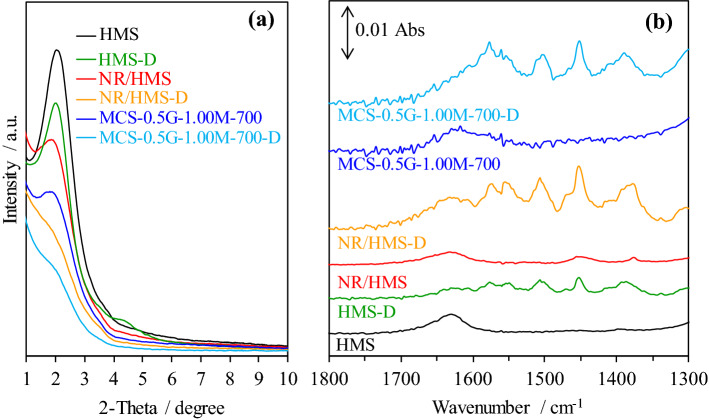
Representative (**a**) XRD patterns and (**b**) FT-IR spectra of HMS, NR/HMS, and MCS carriers with and without diclofenac loading.

HMS-D, NR/HMS-D, and MCS-0.5G-1.00M-700-D were characterized by various techniques to assure successful diclofenac loading on these carriers. The presence of diclofenac decreased the characteristic hexagonal mesostructure (Fig. 9a). The FT-IR spectra of the carriers after drug loading revealed new bands at 1,280–1,350 cm^−1^ and 1,450–1,600 cm^−1^, which corresponded to C–N and aromatic stretching, respectively (Fig. 9b), of the diclofenac molecule^22^. Moreover, the N_2_ physisorption measurement indicated a decrease in *S*_BET_ and *V*_t_ after diclofenac loading (Table 2), which was more pronounced for HMS. Presumably, a larger portion of diclofenac molecules blocked the pore mouth of pure silica carrier, while the hydrophobic properties of NR/HMS and MCS nanocomposites promoted the diffusion and dispersion of diclofenac in mesostructured pores.

**Table 2:** Textural properties of HMS, NR/HMS, and MCS carriers with and without diclofenac loading.

Carriers	*S*_BET_^a^ (m^2^ g^−1^)	*D*_p_^b^ (nm)	*V*_t_^c^ (cm^3^ g^−1^)
HMS	815	2.94	1.80
HMS-D	230	3.52	0.69
NR/HMS	589	2.35	1.40
NR/HMS-D	405	2.30	0.77
MCS-0.5G-1.00M-700	664	2.37	1.41
MCS-0.5G-1.00M-700-D	400	2.17	1.09

^a^BET surface area.

^b^Pore diameter calculated using the BJH method.

^c^Total pore volume.

**Figure 10:**
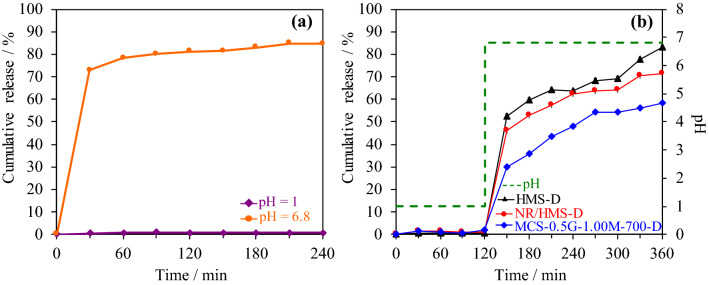
Representative controlled releasing profiles of (**a**) pure diclofenac at pH = 1.0 and 6.8 and (**b**) HMS-D, NR/HMS-D, and MCS-0.5G-1.00M-700-D under simulated gastrointestinal environment.

Diclofenac dissolved very fast in a simulated intestinal environment (pH = 6.8), its cumulative release profile reached an equilibrium of approximately 80% in the first 60 min (Fig. 10a). Using HMS and nanocomposite carriers strongly controlled the sustained release of diclofenac in the intestinal environment (Fig. 10b), which agreed with the previous report by Brovo et al.^60^. The rate of diclofenac released from the three carriers was ranked in the following descending order: HMS-D > NR/HMS-D > MCS-0.5G-100M-700-D, which matched the hydrophobicity trend of these materials (Fig. 8). Then, the cumulative release profiles were fitted with different kinetic models, and the calculated parameters and corresponding correlation coefficients (R^2^) are summarized in Table 3. Diclofenac released from each carrier was best represented by the Higuchi model, which indicated that the release of diclofenac from carriers as a square root of time-dependent process and diffusion control^61^. From the Korsmeyer–Peppas kinetic model, the diffusional exponent (*n*) value was used to characterize different release mechanisms for cylindrical-shaped matrices. HMS-D had *n* higher than 0.89, which indicated that diclofenac release followed the super case II transport^61^, while the smaller *n* values of NR/HMS-D and MCS-0.5G-100M-700-D suggested anomalous diffusion or nonFickian diffusion^61^. These results suggest that the hydrophobicity of NR/HMS and MCS-0.5G-100M-700 slowed down solvent diffusion into their porous structure and the dissolution of loaded diclofenac^62^.

**Table 3:** Kinetic parameters of diclofenac sodium release from HMS-D, NR/HMS-D, and MCS-0.5G-1.00M-700-D.

Sample	Zero order model		First order model		Higuchi model		Korsmeyer–Peppas model			Hixson–Crowell model	
	*K*_*0*_ (% min^−1^)	R^2^	*K*_*1*_ (min^−1^)	R^2^	*K*_*H*_ (% min^−0.5^)	R^2^	*K*_*KP*_ (min^−*n*^)	*n*	R^2^	*K*_*HC*_ (% min^−3^)	R^2^
HMS-D	0.239	0.6649	0.0056	0.8531	5.426	0.9914	0.873	0.90	0.9031	0.010	0.6940
NR/HMS-D	0.212	0.6561	0.0041	0.8146	4.826	0.9901	1.680	0.75	0.9160	0.008	0.5609
MCS-0.5G-1.00M-700-D	0.195	0.7963	0.0032	0.8847	3.878	0.9951	2.338	0.63	0.9160	0.006	0.7002

## Conclusions

MCS nanocomposites with tunable physicochemical properties were successfully prepared from the NR/HMS precursor via carbonization. An increase in the carbonization temperature up to 700 °C resulted in MCS nanocomposites with a well-ordered mesostructure and uniform framework-confined wormhole-like channels; while the use of high concentration of H_2_SO_4_ solution promoted rubber conversion into carbon residues, which resulted in an increased carbon content. An increase in the initial amount of NR in the NR/HMS precursor did not affect the carbon content but lowered the structural and textural properties of resulting MCS materials owing to the limited incorporation and dispersion of rubber molecules into the mesostructure of HMS. MCS nanocomposites were characterized by the high dispersion of graphene oxide-like carbonaceous moieties with different types of surface oxygen-containing groups (C–O, C=O, C–O–O, and O–C=O). The hydrophobic properties of MCS materials were not only determined by the carbon content, but also by the amount of surface silanol and oxygen-functional groups. The NR/HMS precursor and resulting MCS nanocomposites were preliminarily confirmed as potential drug carriers on which a sustained release of drug molecules could be controlled by their surface hydrophobicity. This approach provided a simple strategy for preparing mesoporous nanocomposites with the combined advantageous properties of mesostructured silica and graphene oxide-like carbon for drug delivery applications.

## Materials and methods

### Materials and chemical reagents

TEOS (AR grade, 99%), DDA (AR grade, 98%), sulfuric acid (H_2_SO_4_; AR grade, > 95%) and diclofenac sodium salt (AR grade, ≥ 98%) were purchased from Sigma-Aldrich. The NR (technically specified STR-5L grade) was supplied by the Thai Hua Chumporn Natural Rubber Co., Ltd. (Thailand). Absolute ethanol (AR grade, 99.5%), hydrochloric acid (HCl; AR grade, 37%), and sodium phosphate tribasic dodecahydrate (AR grade, > 98%) were supplied by Merck Ltd. Chemicals, and tetrahydrofuran (THF) (AR grade, 99.5%) were purchased from QRëC. All chemical reagents were used without further purification.

### Synthesis of pure silica HMS

Pure silica HMS was synthesized via the sol–gel process using TEOS as the silica source and DDA as amine template in the presence of THF, as has been previously reported^16^. Typically, DDA was dissolved in THF and deionized water under stirring. To this homogeneous solution, TEOS was added dropwise to obtain gel with the molar composition of 0.05 TEOS: 0.02 DDA: 2.94 H_2_O: 0.56 THF. After stirring at ambient temperature for 30 min, the mixture was aged in an oven at 40 °C for 24 h. The resulting white solid was recovered by filtration, thoroughly washed with ethanol, and dried under vacuum at 60 °C for 12 h. Then, dried solid was calcined at 700 °C for 3 h to remove the organic template.

### Synthesis of NR/HMS and MCS nanocomposites

NR/HMS nanocomposites were prepared via an in situ sol–gel method using THF as the synthesis media. In a typical batch, 0.5 g of the NR sheet was swollen in TEOS at room temperature for 16 h. Then, the swollen NR sheet was dissolved in THF with stirring overnight to obtain a homogeneous solution. Subsequently, DDA was mixed with a rubber solution, followed by the dropwise addition of TEOS with stirring. After 30 min, deionized water was slowly added into the mixture with stirring at 40 °C. The general molar composition of the synthesis mixture was 0.05 TEOS: 0.02 DDA: 2.94 H_2_O: 0.56 THF: 0.005 NR. The obtained gel was aged at 40 °C for 3 days; then, it was precipitated in 50 mL of ethanol. The solid product was recovered by filtration and drying under vacuum at 60 °C for 12 h. During the pretreatment step, as-synthesized NR/HMS was refluxed with an H_2_SO_4_/ethanol solution for 4 h. H_2_SO_4_ adsorbed on the NR/HMS precursor was supposed to work as a catalyst for the conversion of rubber phase into carbonaceous moieties during the subsequent carbonization process.

MCS nanocomposites were prepared by the carbonization of acid-treated NR/HMS materials. Carbonization was performed in a tubular furnace under an argon flow. The temperature was increased at 2 °C min^−1^ to 350, 450, or 700 °C, each of which was maintained for 1 h. The obtained carbon/silica nanocomposites were designated as MCS-*x*G-*y*M-*t*, where *x* represents the initial NR content of the NR/HMS precursor (g), *y* represents the concentration of the H_2_SO_4_/ethanol solution (M), and *t* represents the temperature of carbonization (°C).

### Materials characterization

The thermal decomposition pattern and carbon content of nanocomposites were determined by thermogravimetric analysis (TGA) using a PerkinElmer Pyris Diamond thermogravimetric instrument. The analysis was performed at the heating rate of 10 °C min^−1^ from room temperature to 1,000 °C under dry air flow of 50 mL min^−1^.

Powder XRD was used to investigate the mesostructure ordering of pure silica HMS and nanocomposites. The XRD patterns were recorded on a Bruker D8 ADVANCE diffractometer using Cu Kα radiation generated at 40 kV and 40 mA. The measurement was performed in the 2*θ* range of 0.5–10° with a scanning step of 0.02° and a count time of 1 s. The interplanar spacing of the (100) plane (*d*_100_) was calculated according to Bragg’s equation. The repeating distance (*a*_*0*_) between pore centers of the hexagonal structure was calculated using the formula: *a*_*0*_ = 2*d*_100_/$\sqrt{3}$.

Nitrogen (N_2_) adsorption–desorption measurements were performed at − 196 °C using a Micromeritics ASAP 2020 surface area and porosity analyzer. The sample was degassed at 150 °C for 2 h prior to the measurement. The specific surface area (*S*_BET_) was calculated from the adsorption branch data in the relative pressure (*P/P*_0_) range from 0.05 to 0.3 using the Brunauer–Emmett–Teller (BET) method. The total pore volume (*V*_t_) was obtained from a single point on the adsorption branch at *P/P*_0_ of approximately 0.99. The external surface area (*S*_ext_) and primary mesopore volume (*V*_p_) were estimated using the *t*-plot method. The pore size (*D*_p_) was calculated from the adsorption branch data according to the Barrett–Joyner–Halenda (BJH) equation.

The chemical functional groups of materials were investigated by Fourier transform infrared spectroscopy (FT-IR). A self-supporting disk of each sample (20-mm diameter, 40 mg) was placed at the center of a quartz cell connected to a conventional closed gas-circulation system. The sample was pretreated by evacuation at 150 °C for 1 h to remove adsorbed moisture. The FT-IR spectra were recorded at room temperature in transmission mode on a JASCO FT/IR-4100 spectrometer with a Mercury Cadmium Telluride (MCT) detector with a total of 64 scans over 400–4,000 cm^−1^ at the resolution of 4 cm^−1^.

Raman spectroscopy was used to reveal the carbon structure of MCS nanocomposites. Prior to the analysis, the sample powder was dried at 100 °C overnight to reduce adsorbed moisture. Raman spectra were collected on a JASCO NRS-5100 laser Raman spectrometer with the laser wavelength of 532 nm. Each sample was placed across a glass slide, and each spectrum was recorded for five scans. The surface functional groups of materials were also observed by X-ray photoelectron spectroscopy (XPS) using an ESCA 1700R system with Al Kα1 radiation (1,486.8 eV). The binding energy (BE) for high-resolution C1s, O1s, and Si2p spectra was calibrated by setting C1s at 284.6 eV. The curve-fitting of Raman and XPS spectra was performed with the OriginPro 8.5 software (OriginLab Corporation).

The relative concentration of silica species present in HMS and representative nanocomposites was investigated by solid-state ^29^Si MAS nuclear magnetic resonance spectroscopy (NMR). The ^29^Si NMR MAS spectra and ^13^C cross-polarization (CP) MAS NMR spectra were recorded on a JEOL-ECA600 NMR spectrometer at 79.4 MHz and a sample spinning frequency of 15 kHz. The sample was loaded into a 4-mm zirconium oxide rotor. The recycle delay was 5 s, and the CP contact time was 2 ms. The chemical shifts of ^29^Si NMR MAS spectra and ^13^C CP/MAS NMR spectra are represented in parts per million (ppm) using polydimethylsiloxane as the internal standard. The spectral resolution was sufficient for accurate peak assignments, and the relative peak area of each silica species was obtained by curve-fitting analysis from a series of Gaussian curves using the OriginPro 8.5 software.

The morphological study was performed by field-emission scanning electron microscopy (FE-SEM) using a Hitachi SU5000 instrument operated at 40 kV. The sample powder was dispersed on carbon tape, followed by platinum coating. TEM was used to directly observe the mesoporous structure of materials. The TEM images were recorded at the magnification of 100,000× using a JEOL JEM-2010F transmission electron microscope operated at an accelerating voltage of 200 kV.

The hydrophobicity of HMS and representative nanocomposites was determined using the H_2_O adsorption measurement. The adsorption experiment was conducted at 25 °C using a BEL Japan BELSORP-max instrument. The sample weight was measured exactly after pretreatment at 150 °C for 2 h. The monolayer adsorbed volume (*V*_m_) of H_2_O was determined from the analysis of adsorption data at *P/P*_0_ below 0.2.

The formation of mesophase during the synthesis of NR/HMS was characterized by SAXS. The SAXS measurement was performed on a beamline 1.3 W using 1.2 GeV synchrotron light source at Siam Photon Laboratory, Synchrotron Light Research Institute, Thailand. The synchrotron light originating from a bending magnet was monochromatized using a double multilayer monochromator to provide an X-ray energy of 9 keV. A toroidal mirror was used to focus X-rays to the sample position. The experimental station was equipped with a charge-coupled device^63^.

### Loading of diclofenac sodium onto carriers

Diclofenac was loaded onto HMS, NR/HMS, and MCS-0.5G-1.00 M-700 by incipient-wetness impregnation. A diclofenac solution (50 ppm) was prepared by dissolving diclofenac sodium salt in ethanol under stirring. Subsequently, diclofenac solution was added dropwise onto the carrier (solid:liquid mass ratio of 1:2), followed by drying at 55 °C for 2 h. The obtained diclofenac loaded carriers were designated as HMS-D, NR/HMS-D, and MCS-0.5G-1.00M-700-D.

### In vitro diclofenac release studies

The controlled release of diclofenac assay was performed as batch experiments using a shake-flask method. The phosphate buffer solution was prepared by dissolving sodium phosphate tribasic dodecahydrate in water. In a typical batch, 100 mg of diclofenac was added into HCl (pH 1.0) or phosphate buffer (pH 6.8) solutions. The mixture was agitated in a rotary shaker at 37 ± 0.5 °C at 100 rpm. The drug released kinetics was evaluated by measuring the diclofenac concentration at different time intervals. The aliquot of liquid (5 mL) taken from the mixture was filtered through a polytetrafluoroethylene syringe filter, and the filtrate was diluted with 20 mL of buffer. Then, the concentration of released diclofenac was measured by a UV-spectrophotometer (Biochrom Libra S22, UK) at the wavelength of 276 nm. In the simulated gastrointestinal environment, the release of diclofenac from carriers (100 mg diclofenac loaded on carrier) was conducted in the HCl solution (pH 1) for 2 h. Subsequently, the mixture pH was adjusted to 6.8 using a phosphate buffer solution, and maintained for 4 h. Then, the experimental data were evaluated for the possible controlled releasing process using the zero order, first order, Higuchi, Korsmeyer–Peppas and Hixson–Crowell kinetic models. The equations of these kinetic models are described in SI.

The percentages of cumulative release were calculated according to Eq. (1):

1 $$ {\text{Cumulative release}} = \left( {C_{t} {/}C_{0} } \right) \times {1}00\% $$

where *C*_*t*_ is the concentration of drug released at any time t, and *C*_*0*_ is the initial concentration of drug at time t = 0 (mg_diclofenac_/kg_carier_).

## Supplementary information

Supplementary Information.
