# Supplementary material for: Natural rubber as a renewable carbon source for mesoporous carbon/silica nanocomposites
Source: Sci Rep. 2020 Jul 31;10:12977. doi: 10.1038/s41598-020-69963-3 (PMC7395082; doi:10.1038/s41598-020-69963-3)
Supplement: Supplementary file 1 — Supplementary Information. [file 41598_2020_69963_MOESM1_ESM.docx]

**Supplementary Information**

**NATURAL RUBBER AS A RENEWABLE CARBON SOURCE FOR MESOPOROUS CARBON/SILICA NANOCOMPOSITES**

**Satit Yousatit^1,2^, Hannarong Pitayachinchot^1^, Apinya Wijitrat^1^,**

**Supphathee Chaowamalee^1,2,3^, Sakdinun Nuntang^4^, Siriwat Soontaranon^5^,**

**Supagorn Rugmai^5^, Toshiyuki Yokoi^6^, Chawalit Ngamcharussrivichai^1,2,3,*^**

^1^*Department of Chemical Technology, Faculty of Science, Chulalongkorn University, Pathumwan, Bangkok 10330, Thailand*

^2^*Center of Excellence in Catalysis for Bioenergy and Renewable Chemicals (CBRC),*

*Faculty of Science, Chulalongkorn University, Pathumwan, Bangkok 10330, Thailand*

^3^*Center of Excellence on Petrochemical and Materials Technology (PETROMAT), Chulalongkorn University, Pathumwan, Bangkok 10330, Thailand*

^4^*Industrial Chemistry and Textile Technology Programme, Faculty of Science,*

*Maejo University, Chiang Mai 50290, Thailand*

^5^*Synchrotron Light Research Institute (SLRI)*, *Nakhon Ratchasima 30000, Thailand*

^6^*Nanospace Catalysis Unit, Innovative Research Institute, Tokyo Institute of Technology,*

*4259 Nagatsuta, Midori-ku, Yokohama 226-8503, Japan*

^*^ Corresponding author. Tel.: +66 2218 7528; fax: +66 2255 5831.

*E-mail address:* [Chawalit.Ng@Chula.ac.th](mailto:Chawalit.Ng@Chula.ac.th)

**Table S1** Raman spectroscopy results of the pure silica HMS, NR/HMS precursor, and MCS nanocomposites

| Sample | Relative area (%) | | | | | I_D1_/(I_D1_+I_G_+I_D2_) |
| --- | --- | --- | --- | --- | --- | --- |
|  | D_4_ | D_1_ | D_3_ | G | D_2_ |  |
| HMS | - | - | - | - | - | - |
| NR/HMS | - | - | - | - | - | - |
| MCS-0.5G-0.05M | 5.86 | 46.27 | 10.05 | 21.88 | 15.93 | 0.55 |
| MCS-0.5G-0.50M | 6.30 | 43.58 | 12.04 | 21.58 | 16.51 | 0.53 |
| MCS-0.5G-1.00M | 7.08 | 43.66 | 12.43 | 20.90 | 15.92 | 0.54 |
| MCS-0.5G-1.50M | 7.16 | 43.93 | 13.66 | 20.50 | 14.75 | 0.55 |
| MCS-0.5G-2.00M | 7.25 | 40.75 | 16.32 | 18.68 | 16.99 | 0.53 |
| MCS-1.0G-1.00M | 7.87 | 44.96 | 9.26 | 22.79 | 15.12 | 0.54 |
| MCS-1.5G-1.00M | 8.18 | 48.23 | 7.46 | 24.96 | 11.17 | 0.57 |

**Table S2** XPS results of the pure silica HMS, NR/HMS precursor, and MCS nanocomposites

| Atomic orbital | Bond type | HMS |  |  | NR/HMS | |  | MCS-0.5G-1.00M-700 | |  | MCS-0.5G-2.00M-700 | |  | MCS-1.5G-1.00M-700 | |
| --- | --- | --- | --- | --- | --- | --- | --- | --- | --- | --- | --- | --- | --- | --- | --- |
|  |  | BE (eV) | Conc.^a^ (%) |  | BE (eV) | Conc.^a^ (%) |  | BE  (eV) | Conc.^a^ (%) |  | BE  (eV) | Conc.^a^ (%) |  | BE  (eV) | Conc.^a^ (%) |
| C1s | C=C | - | - |  | 282.9 | 3.25 |  | 283.6 | 6.51 |  | 283.7 | 7.80 |  | 284.5 | 6.97 |
|  | C−C/ C−H | 285.5 | 3.23 |  | 284.8 | 11.25 |  |  |  |  |  |  |  |  |  |
|  | C−O/ C−O−C | 286.7 | 0.91 |  | 285.8 | 2.88 |  | 285.3 | 1.26 |  | 285.7 | 3.15 |  | 285.7 | 1.33 |
|  | C=O | - | - |  | 287.2 | 1.41 |  | 287.2 | 0.54 |  | 287.3 | 1.57 |  | 287.3 | 0.43 |
|  | O=C−O | - | - |  | - | - |  | 289.0 | 0.07 |  | 289.0 | 0.20 |  | 289.1 | 0.11 |
| Si2p | Si−O | 104.5 | 15.13 |  | 104.4 | 12.26 |  | 104.1 | 14.04 |  | 104.0 | 14.22 |  | 104.0 | 13.62 |
| O1s | O=C | - | - |  | 529.8 | 3.74 |  | 530.3 | 1.75 |  | 530.5 | 5.50 |  | 530.2 | 1.57 |
|  | O−C | 531.4 | 6.46 |  | 531.8 | 7.82 |  | 531.7 | 4.74 |  | 532.0 | 10.44 |  | 531.5 | 5.47 |
|  | Si−O−Si | 536.4 | 51.35 |  | 533.3 | 48.85 |  | 533.0 | 68.63 |  | 534.4 | 54.80 |  | 533.5 | 67.65 |
|  | Si−O−H | 537.5 | 22.93 |  | 534.2 | 8.08 |  | 534.7 | 2.47 |  | 535.7 | 2.34 |  | 535.9 | 2.84 |
|  | O−H ^b^ | - | - |  | 535.9 | 0.46 |  | - | - |  | - | - |  | - | - |

^a^ Atomic concentration. ^b^ As water.

**Fig. S1** Representative Raman spectra of MCS-0.5G-1.00M-700, MCS-0.5G-2.00M-700, and MCS-1.5G-1.00M-700 at high Raman shift region.


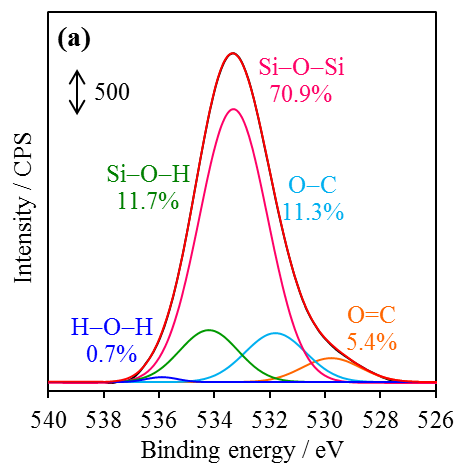

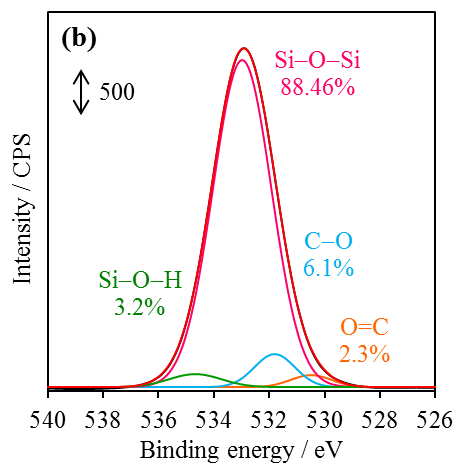

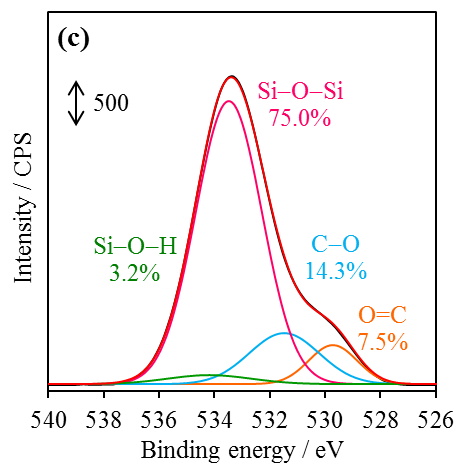

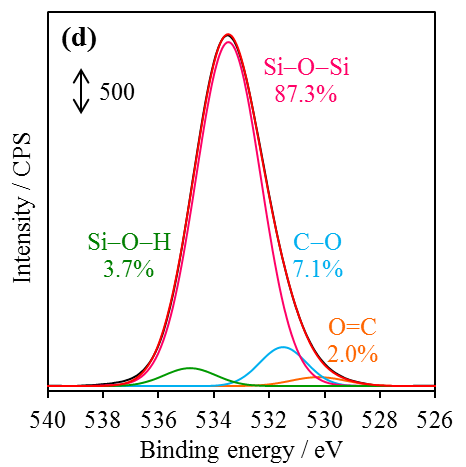


**Fig. S2** Representative O1s XPS spectra of (A) NR/HMS, (B) MCS-0.5G-1.00M-700, (C) MCS-0.5G-2.00M-700, and (D) MCS-1.5G-1.00M-700.

***Release kinetic models***

The experimental data were evaluated for the mechanism of drug release from the carrier using the zero order, first order, Higuchi, Korsmeyer−peppas and Hixson−crowell kinetic models. The zero order equation [1] was defined as in Eq. (S1);

*Q_t_* = *Q_0_* + *K_0_t* (S1)

where *Q_0_* and *Q_t_* are the cumulative percentage released at initial time and at any given time (t), respectively, and *K_0_* is the zero-order rate constant, which was determined from the plots of  *Q_t_* versus *t*.

The first order equation [1] can be expressed as in Eq. (S2);

ln *Q_t_* = ln *Q_0_* - *K_1_t* (S2)

where *K_1_* is the first order rate equation expressed in time^-1^ or per hour, *Q_0_* is the initial cumulative percentage of the drug and *Q_t_* is the cumulative percentage remaining at any time (t). The slope of the plot of ln *Q_t_* versus *t* gives the first order rate constant.

The Higuchi model [1] can be described as Eq. (S3);

*Q_t_* = *K_H_* × *t*^1/2^ (S3)

where *Q_t_* is the cumulative percentage released at any given time (t) and *K_H_* is the Higuchi dissolution constant, which was evaluated from the plots of *Q_t_* versus *t*^1/2^.

The linear form of Korsmeyer−peppas model [1] can be described as Eq. (S4);

ln (*M_t_ / M_∞_*) = ln *K_KP_* + *n* ln *t* (S4)

where *M_t_ / M_∞_* is a fraction of drug released at time t, *M_t_* and *M*_∞_ are the amount of drug released at any time (t) and at equilibrium (mg_diclofenac_/kg_carier_), *n* is the diffusional exponent or drug release exponent (dimensionless) and *K*_KP_ is the Korsmeyer release rate constant (min^-^*^n^*). To study release kinetics a graph is plotted between ln (*M_t_ / M_∞_*) versus ln *t*. Hence, *n* value is used to characterize different release mechanisms as given in tabular form (Table S3) for cylindrical shaped matrices.

**Table S3** Interpretation of release mechanisms model for cylindrical shaped matrices [1,2].

| Release exponent (*n*) | Release mechanism model | Rate as a function of time |
| --- | --- | --- |
| 0.5 | Fickian diffusion | *t*^0.5^ |
| 0.45 < *n* < 0.89 | Anomalous transport or  Non-Fickian transport | *t*^0.45 <^ *^n^* ^< 0.89^ |
| 0.89 | Case II transport | *t*^0.89^ |
| *n* > 0.89 | Super case II transport | *t^n^* ^> 0.89^ |

The linear form of Hixson−crowell model [1] can be described as Eq. (S5);

*Q_0_*^1/3^ − *Q_t_*^1/3^ = *K_HC_* × *t* (S5)

where *Q_0_* is the initial percentage of the drug in carrier and *Q_t_* is the cumulative percentage drug remaining in carrier at any time (t), respectively, and *K_HC_* is the Hixson-Crowell constant describing surface volume relation (% min^-3^), which was examined from the plots of
*Q_t_*^1/3^ versus *t*.

**References**

[1] Baishya, H. Application of mathematical models in drug release kinetics of Carbidopa and Levodopa ER tablets. *J. Dev. Drugs* **06**, 2; 10.4172/2329-6631.1000171 (2017).

[2] Marcos, L. B., Mathematical models of drug release in *Strategies to Modify the Drug Release from Pharmaceutical Systems* (ed. Marcos, L. B.) 63–86 (Woodhead Publishing, 2015).
